# Effects of concurrent training on glycemic and vascular parameters among patients with T2DM-associated Peripheral Artery Disease

**DOI:** 10.12669/pjms.40.8.9045

**Published:** 2024-09

**Authors:** Uroosa Amin, Qurat-ul-Ain Adnan, Tauseef Ahmad

**Affiliations:** 1 Uroosa Amin, Ziauddin College of Rehabilitation Sciences, Ziauddin University, Khayaban-e-Saadi Road, Boat Basin, Karachi, Pakistan; 2 Qurat-ul-Ain Adnan, PhD (Scholar) Vice Principal, Assistant Professor, Ziauddin College of Rehabilitation Sciences, Ziauddin University, Khayaban-e-Saadi Road, Boat Basin, Karachi, Pakistan; 3 Dr. Tauseef Ahmad, FCPS Ziauddin Hospital, Clifton, Block-5, Karachi, Pakistan

**Keywords:** Ankle-brachial Indices, Diabetic vascular Complication, Glycated Hemoglobin, Peripheral Artery Disease, Type 2 Diabetes Mellitus

## Abstract

**Objective:**

To evaluate the effects of CT to improve HbA1C and ABI among the T2DM-associated PAD population.

**Methods:**

A randomized, single-blinded, two-arm trial was conducted at the Department of Rehabilitation Sciences of Dr. Ziauddin Hospital in Karachi between July to September 2023. A total of 80 T2DM-associated PAD patients were included and randomly divided into Experimental Group (n=40) and Control Group (n=40), using the sealed envelope method. Experimental group patients received Concurrent Training (CT), whereas Control Group patients underwent Aerobic Training (AT) for 12 weeks. Both groups received thirty-minute sessions three times a week that was progressed to 60 minutes over 12 weeks. HbA1C and ABI were measured at baseline and after 12 weeks.

**Results:**

Analysis revealed an average age of 46.75±3.59 and the duration of T2DM for developing PAD is 14.82±2.23 on average. Findings revealed that both training groups were significantly effective (p<0.000) at 95% CI in improving glycemic and vascular parameters after 12 weeks. Subsequently, findings showed that the CT group showed more significant improvement than AT group in improving HbA1C for glycemic control (p=0.002, CT: pre: 9.53±1.406, post: 7.81±0.81, AT: pre: 8.74±0.908, post: 8.15±0.83) and ABI for systemic blood flow (p=0.0001, CT: pre: 0.84±0.03, post: 0.94±0.03, AT: pre: 0.82±0.02, post: 0.86±0.02).

**Conclusion:**

CT showed a two-fold improvement in glycemic control and arterial blood flow than AT group, which represents that CT is an effective therapeutic approach for T2DM-associated Fontain’s stage IIa PAD rehabilitation.

## INTRODUCTION

Globally, the diabetes epidemic has increased in the general population over the last few decades, which is quite alarming.^1^ Type-II diabetes mellitus (T2DM) is highly prevalent (>90%), affecting 537 million adults worldwide and is predicted to affect 783 million adults by 2045.^1^ According to the International Diabetes Federation (IDF) regional distribution, Pakistan is included in Middle East and North Africa region with the highest prevalence of Diabetes among adults and ranks the topmost which is predicted to be 135 million by 2045.^1^ Eventually, diabetes-specific sequelae increased the need for healthcare services and financial costs.^2^ Majority of the diabetes burden is associated with macrovascular complications, such as coronary heart diseases (CHDs), stroke, and peripheral vascular disease.^2^ Insulin resistance increases as a result of altered body composition, gradual decline in physical activity, which adversely affects their QOL.^3^ Remarkably, adults (aged 45/>) with T2DM are at an increased risk of physical disability by 50–80%.^4^ Despite higher prevalence in low-middle-income countries (LMICs), population-based studies and data systems for diagnosing and assessing Diabetes-related complications in LMICs are scarce, resulting in additional challenges.^2^

International guidelines for managing T2DM recommend exercise as a foundation but still it is neglected.^5^ Moreover, delayed diagnosis adversely affects the blood vasculature and leads to arteriosclerosis and atherosclerosis. Therefore, it’s crucial to prevent acute and long-term vascular complications through early detection and optimization of glycemic control in clinical settings. WHO reported an increase of 3% Diabetes-related death rates from 2000 to 2019, whereas in LMICs by 13%.^6^

Diabetes-related vascular complications, i.e. Peripheral arterial disease (PAD) commonly affects the lower extremity (Aortoiliac to pedal arteries) than the upper extremity vasculature.^7^ PAD’s pathogenesis is a vicious cycle that has an atherosclerotic (clot/ thrombus) obstruction-induced blood flow restriction which results in ischemia and endothelial dysfunction that causes skeletal muscle fiber denervation, atrophy, and altered myosin expression, ultimately alters aerobic muscle metabolism. Consequently, poor aerobic capacity decreases muscle strength, & endurance that impairs walking and QOL which can advance the disease severity by further deconditioning and worsening of risk factors.^8^ American Heart Association’s (AHA) Scientific Statement highlighted the PAD as an underdiagnosed and undertreated condition and frequency of 5% in the age of 40-44. Globally, epidemiological studies have reported that 20% to 26% of Type-II DM has PAD, which leads to poor health consequences.^8^ In Pakistan, the prevalence of PAD was 31.5%.^9^ Despite this, only 6% of primary-care physicians were aware of PAD’s prevalence, severity, and management guidelines, as opposed to the average screening rate of < two-thirds for the disease state.^10^ The duration of Diabetes is strongly associated with the development of PAD, and the frequency of PAD is 40% after 10 years of T2DM which is assessed by intermittent claudication.^11^ Furthermore, Ankle Brachial Index is used to asses vascular status of PAD patients, in which ratio of systolic pressures of brachial and dorsalis pedis artery is measured. ABI of 0.81-0.90 has a two-fold higher mortality rate than usual. The combination of pharmacotherapy and exercise is considered the standardized care for T2DM patients with PAD.^9^

In addition, American College of Sports Medicine (ACSM) and AHA strongly recommended supervised exercise as the first-line management of PAD patients. Revascularization is only recommended in situations resistant to medical therapy and exercise.^9^ Exercise training can delay the emergence of T2DM & prevents complications, thereby maximize the therapeutic efficacy among Diabetes related PAD management.^11^ Traditionally, aerobic exercise in diabetic patients positively impacts metabolic factors, i.e., lipid profile, subcutaneous fat, and blood glucose.^12^ Moreover, modified aerobic exercise of sufficient duration and intensity can lower Glycosylated hemoglobin glycosylated (HbA1c), enhance muscle metabolism, and minimize oxidative stress.^12^

Furthermore, Resistance training significantly improved the ABI, ambulatory arterial stiffness index (AASI), and the endothelin-1, systolic, and diastolic blood pressure after 12 weeks.^13^ Due to the compensatory mechanisms of both types of exercise, combined exercises can have twice the effects on patients with Diabetes and PAD at different intensities.^14^ Moreover, aerobic and resistance training within the same training session in term of concurrent training (CT) for 8 weeks was observed to be more effective in improving glycemic index, muscle volume and Myogenic factor.^15^ Limited evidence about appropriate exercises for individual patient’s characteristics and the beneficial effects of CT on glycemic and vascular parameters compared to isolated aerobic exercise among diabetes patients with PAD with Asian demographics highlights the need to evaluate it. Therefore, this study aimed to effectively determine appropriate exercise training by integrating individualized exercise protocol into the clinical settings; thereby reducing risk factors for T2DM-associated PAD patients.

## METHODS

A two-arm, single-blinded, randomized controlled trial was conducted at Department of Rehabilitation sciences of a well-equipped tertiary care facility, Dr. Ziauddin Hospital and data was collected from July to September 2023.

### Ethical Approval and Trial Registration:

This trial has followed the standards of the Declaration of Helsinki for human research subjects, approved by the Ethical Review Committee of Ziauddin University (Protocol Ref#7170523HSPAT) and registered in the Clinical Trials Registry (NCT06028399).

### Inclusion & Exclusion Criteria:

Before induction, all patients received verbal and written information about the study. Afterwards, their voluntary consent was obtained. Participants were included who were diagnosed with T2DM >10 years duration, aged 40-50 years, Baseline HbA1C (>6.6%), Intermittent claudication (Fontaine Stage-II(a)), PAD (ABI: < 0.9 at rest), had BP < 160/105 mmHg, and Sedentary lifestyle by IPAQ-SF (category-1). Patients were excluded if they were smokers, insulin-dependent, complicated CVDs (e.g. unstable cardiopulmonary symptoms, ischemic heart failure, or chronic kidney disease) and cancer, any major surgery or revascularization procedure within the previous one year, history of severe arthritis, diabetic foot (ulceration or gangrene), stroke patients, WHO-Asian classifies BMI = >30 (obesity class II) , unable to follow the intervention protocol or visit three times/week for exercise, and revocation to consent.

The confidentiality of each patient was respected with rights to withdraw from participating in the trial. Eighty T2DM-associated PAD patients were assigned to intervention groups randomly using a Sequentially Numbered, Opaque Sealed Envelope (SNOSE) technique. Subsequently, patients were randomly allocated into Experimental Group (EG n=40) and Control Group (CG n=40). Once patients get allocated, proper education and understanding about their respective group exercises, rest intervals, treadmill walk, Thera band usage with an acquaintance of band color and criteria of progression was given.

Outcome measures, HbA1C and ABI were measured at baseline and after 12 weeks. Both groups had 36 sessions of their respective regimen under the supervision of experienced therapists, each lasting 30-60 minutes with the frequency of three times per week for 12 weeks. The HbA1c is a gold standard for assessing glycemic control for 2-3 months with an excellent reliability and validity (Sn= 0.8, Sp=0.9, AUROC = 0.9).^15^ ABI is a non-invasive clinical measure for determining the presence and severity of PAD and Doppler ABI has Sn=80.3% and Sp=78.1%.^16^ An ABI of 0.9 is considered as PAD evaluated by a portable Doppler Ultrasound with a 5MHz probe. The higher systolic pressures of the right and left brachial, divided by higher pressures of dorsalis pedis arteries to obtain ABI value. For training, both groups performed warm-up exercises for 10 - 12 minutes first, followed by intervention protocol and then cool-down exercises for 10 - 12 minutes.

During the intervention period, patients were assessed for Heart rate, blood pressure, and random blood sugar before and after each session for 12 weeks’ duration, and perceived exertion status by using Borg’s rate of perceived exertion scale (6-20) for analyzing the exercise intensity. The targeted intensity for enrolled participants was 13-15. EG group performed CT (aerobic with resistance training using an elastic resistance band within the same session) under the supervision of the physical therapist following appropriate rest intervals and weekly progression as illustrated in Table-I. The exercise intervention and weekly progression of CG is presented in Table-II.

**Table-I:** FITT Protocol for Concurrent Training (CT) – Experimental Group

CT (Aerobic Training Weekly Progression)										

Weeks	F		I		T		T		P- PATTERN	
1-2	3 days per week		40% THR			15 mins.	Aerobic (treadmill walking)		Seated rest intervals of 2-3 minutes as symptoms occur and resume as symptoms alleviated.	
3-4			40% THR		20 mins.					
5-6			50% THR		25 mins.					
7-8			50% THR		30 mins.					
9-10			60% THR		35 mins.					
11-12			60% THR		40 mins.					

***CT (Resistance Training Weekly Progression)***										

***Weeks***	***F***	***I***		***T***		***T***		***V***		***P***

1-2	3 days per week	60% 1-RM		Time to complete the set per major muscle.		Elastic resistance band exercises As shown in figures. 2 Sets of 10 reps. 3 sets of 8 reps.		1 set of 12 reps.		Rest interval of 2 minutes between each set of reps.
3-4										
5-6		70% 1-RM								
7-8										
9-10		80% 1-RM								
11-12										


F-frequency, I-intensity, T-type, T-time, THR = HRmax x % intensity, and HRmax = 220 – age, 1RM will be calculated for intensity progression as per the patient’s capability.

**Table-II:** FITT Protocol of Aerobic Training (AT) – Control Group

AT (Weekly Progression)					

Weeks	F	I	T	T	P- PATTERN
1-2	3 days per week	40% THR	30 mins.	Aerobic (treadmill walking)	Rest interval of 2-3 min. as symptoms occurs & resume after complete alleviation of symptoms.
3-4		40% THR	35 mins.		
5-6		50% THR	40 mins.		
7-8		50% THR	45 mins.		
9-10		60% THR	50 mins.		
11-12		60% THR	60 mins.		

F-frequency, I-intensity, T-type, T-time, THR = HRmax x % intensity, and HRmax = 220 – age.

After the completion of 12 weeks of intervention, all patients were assessed for HbA1C and ABI and documented for further data analysis. Exercise termination criteria includes the onset of Chest pain, Shortness of breath (Borg’s scale >15), Hemodynamic imbalance during exercise is caused by limited perfusion (Pale appearance, cool skin, Diaphoresis (sweating), Physical exertion, altered pulse) and Low blood pressure (very late sign), on the patient’s request to stop exercise, Ischemic limb pain that is not alleviated after 10 minutes.^13^

### Statistical Analysis:

Data entered and analyzed on SPSS Statistical Software, version 22. The participants’ demographic data was determined through descriptive statistics in frequency, mean and standard deviations. For inferential statistics, if the data is normally distributed therefore Paired T-test was applied for within the group analysis for both CT and AT groups on quantitative measures, i.e., HBA1C and ABI. At the same time, Independent T-test was employed between both groups to determine post-mean values on similar outcome measures. Moreover, p<0.05 was considered significant.

## RESULTS

**Fig.1:**
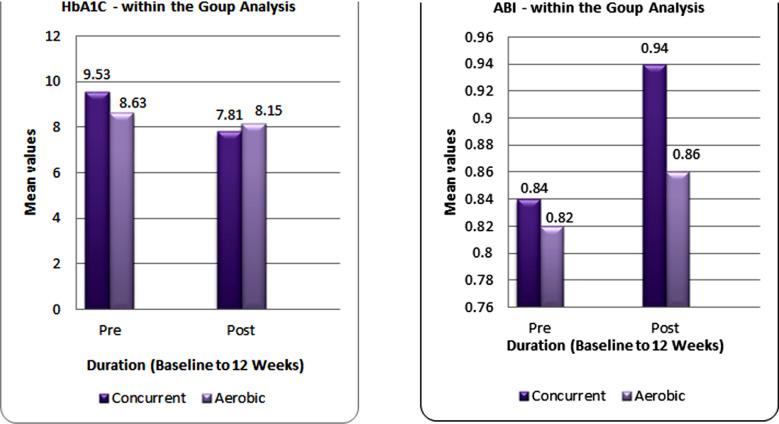
Baseline and 12 weeks Comparison on HbA1C and ABI.

Analysis revealed an average age of 46.75±3.59 for CT and 47.05±2.05 for the AT group and the duration of T2DM for developing PAD is 14.82±2.23 for CT, whereas 12.73±1.92 for AT. Statistically, a paired sample t-test was employed, assuming the normally distributed data. Both groups significantly improved the HbA1C after 12 weeks intervention (p<0.05). To ensure comprehensive hypothesis testing of normally distributed data, findings revealed that CT substantially improved HbA1C (p=0.002) and ABI (p=0.0001) more than at 95% Confidence interval for observed values. Statistically, the findings of HbA1c revealed that the AT group improved with a mean difference (MD) of 0.66, whereas the CT group showed 1.72. For evaluating systematic blood flow, ABI’s findings suggested that the AT group improved with a MD of 0.038 whereas the CT group showed a more significant MD of 0.099. These findings represent the considerable effects of CT over AT. An insight into the comparative effects of CT and AT in improving HbA1C and ABI is further explained in Table-III and Fig.1.

**Table-III:** Comparison of Mean, Standard Deviation and P-Value of both group for HbA1C and ABI.

Comparison of both group for HbA1C and ABI						

variables	Pre Mean±S.D CT Group (n=40)	Pre Mean±S.D AT Group (n=40)	p-value	Post Mean±S.D CT Group (n=40)	Post Mean±S.D CT Group (n=40)	p-value
HbA1C	9.53±1.406	8.74±0.908	0.00001	7.81±0.81	8.15±0.83	0.002
ABI	0.84±0.03	0.82±0.02	0.00001	0.94±0.029	0.86±0.021	0.0001

MD-Mean Difference, CT- Concurrent Training, AT- Aerobic Training, CI-Confidence Interval.

## DISCUSSION

The current study’s results demonstrated that, when compared to the T2DM-PAD patients in the conventional group (n=40) who received treatment for the same amount of time with aerobic exercise, the experimental group (n=40) T2DM-PAD patients, who were treated three times a week for 12 weeks with a CT Regimen, improved their HbA1C and ABI more effectively. Numerous national and international guidelines emphasize the evidence for walking interventions. However, other exercise modes, i.e. cycling, may be beneficial in reducing symptoms among individuals with T2DM-PAD.^17^ Higher plasma nitrite concentrations and disease improvement are potentially attributed to increased muscular perfusion more efficiently after performing Supervised exercise training (SET).^18^ Supervised Exercise on treadmill is highly researched mode to manage PAD patients.

In particular, both study groups revealed statistically noteworthy improvement in glycemic and vascular parameters. However, CT was superior to AT, demonstrating an apparent and clinically significant improvement in recovery of T2DM-PAD patients. Similarly, 12-week concurrent (CROS) or aerobic (AER) exercise among middle-aged T2DM patients showed that CROS group responded to HbA1c better than the AER group (CROS= 5.2±1.3, AER= 7.8±1.6%; p≤0.049).^19^ Similarly, our study’s key findings proved that 12-week CT for T2DM-PAD patients improves HbA1C more than AT, concerning Fontain’s stage 2 of PAD among T2DM patients for more than ten years. The Target heart rate and 1RM were used for the various intensities of the recommended training programs for each group. The CT method included resistance using resistance band and aerobic activities through treadmill.

Additionally, 52 overweight females with T2DM who performed either sprint interval training (SIT) or concurrent training (CCT) for ten weeks showed a considerable improvement in both groups’ HbA1C (SIT=9.64 ± 1.07, CCT= 9.49 ± 0.85) (P 0.001). However, SIT had a more significant stimulatory effect. This trial has multiple drawbacks, including non-assessed SIT methods for safety in T2DM females, smaller size, and a considerable dropout rate.^20^ In contrast; our study’s key findings proved that 12-week CT for individuals with T2DM- PAD improves HbA1C more than AT. This heterogeneous finding in our study is due to the T2DM patients who presented with macrovascular complication i.e. PAD and functional impairments. Therefore, SIT could lead to adverse consequences. For special population considerations, ACSM’s guidelines were prioritized in the current study, which recommends moderate-intensity exercise that gradually progresses to vigorous intensity.

Although there are many studies supporting the potential advantages of CT in maintaining glucose homeostasis for T2DM patients, which is still unknown how this exercise modality may help prevent T2DM-related comorbidities. Following 16 weeks of counseling and CT (MICT - 40 minutes and RT at Borg 12-16) in women Fasting Plasma Glucose decreased by 4.07mg/dL.^21^ Many biological mechanisms play a vital role to show the preventive effect of CT to combat the risk of cardio-metabolic illness. Muscle contractions can cause a quadrupled increase in the screening and transfer the location of Glucose transporter protein-4 (GLUT-4) through an increase in cytoplasmic calcium concentration or membrane depolarization to plasma membrane.

In the latest research, two CT protocols were used for 20 weeks: HIIT 60 seconds of maximal-intensity cycling exercise followed by 1-2 minutes of recovery in passive manner, intensity estimated using the Borg scale for RT exercises (work-rest ratio=1:1) found that total cholesterol reduced by 15mg/dL and triglyceride improved by 10mg/dL. This suggests that CT can potentially improve risk factors in women with Metabolic syndrome.^22^ Skeletal muscle system is the most extensive organ system which accounts for around 40% of an adult’s body weight. It is crucial for metabolism, soft tissue support, temperature and glucose balance, mobility, posture, and temperature regulation. Over 80% of the postprandial glucose absorption from an oral glucose load occurs in skeletal muscle, therefore being essential for glucose clearance.^23^

Long-term vascular problems in T2DM patients can be decreased through CT at different intensities and by improving peripheral artery stiffness indexes and dispensability coefficients. To ascertain the effects of a 12 week CT Regimen (twice weekly) among 41 metabolic syndrome patients involved 50 minutes of strength training (40-70% 1RM) and 40 minutes of walking activities (70-85% HR_max_), which increased muscular strength in the CT (p = 0.001) along with decreasing biguanide consumption (p = 0.002).^24^ Our study’s key findings proved that 12-week Regimen for individuals with T2DM-PAD, which gradually progress the duration, volume and intensity in both groups, improved ABI in the CT group more significantly than AT. Combined training with high intensity is evident to be superior to aerobic training for enhancing lower limb muscle strength and peak oxygen uptake up to 38% and 18%, respectively, without any negative effects from the two distinct exercise modalities during the same session.

Duration and manner of exercises employed in resistance training are crucial factors to produce beneficial results.^25^ These findings were heterogeneous to our study’s findings because it considered a smaller sample size and unidentified age range at the time of inclusion, and the findings have revealed age on average is >60 years. Furthermore, as they did not use the 1RM test to measure training load, which may have overestimated exercise intensity, the resistance training dose-response was minimal. As a result of synergistic effects of aerobic with resistance training, we hypothesized that CT would offer several additional benefits. Our hypothesis was confirmed by the finding of ABI, which significantly improved by the CT group more than AT. Our results show that a CT intervention reverses the chronic adverse consequences regarding systemic blood flow restriction in adults with hyperglycemia and lower extremity vascular atherosclerotic occlusion.

### Limitations:

The first constraint was participant’s refusal to participate due to lack of time. Furthermore, substantial variability in the subjects’ physical activity level and comorbidities at baseline may have influenced their recovery patterns. In addition, findings cannot apply to patients with T1DM or gestational diabetes or who have more asymptomatic or severe baseline vascular dysfunction which needs to be further evaluated.

Furthermore, although both interventions matched dosage, Concurrent exercise is only compared with aerobic exercise. Future research might attempt to evaluate the effectiveness of different modes of exercise with varying intensities and volumes to reduce any potential confounders. To address the limitations of this study, further large-scale investigations, including insulin-dependent DM, smokers, different PAD Fontain’s stages and follow-ups are warranted for generalization of results in different diabetes types and clinical settings.

## CONCLUSION

Concurrent training (CT) as an accessible, risk-free, and potentially successful therapeutic strategy for individuals with type-II DM-associated PAD highlighted more significant improvements in glycemic control, and vascular resistance. As a result, CT with an elastic resistance Thera band is a more economical, clinically effective, and statistically significant intervention.

### Authors Contribution:

**UA** and **QA:** Conceived, designed and did statistical analysis & editing of manuscript, is responsible for integrity of research. **UA**, **QA**, and **TA:** Did data collection and manuscript writing. **QA** and **TA:** Reviewed the data for critical analysis and final approval of manuscript.
